# Clinic and Ultrasound Evaluation of Suction Drainage in Total Knee Arthroplasty Procedure: A Multicentric Prospective Trial

**DOI:** 10.1007/s43465-025-01501-7

**Published:** 2025-07-19

**Authors:** Alessandro Smimmo, Mattia Basilico, Nadia Bonfiglio, Pasquale Ruberto, Raffaele Vitiello, Guido Bocchino, Angelo Carosini, Greta Tanzi Germani, Matteo Salvini, Emidio Di Gialleonardo, Giuseppe Malerba, Vincenzo De Santis

**Affiliations:** 1 Department of Aging, Neurological, Orthopedic and Head-Neck Sciences, Fondazione Policlinico Universitario Agostino Gemelli IRCCS, Rome, Italy; 2 Università Cattolica Del Sacro Cuore, Rome, Italy; 3 Mater Olbia Hospital, Olbia, Italy; 4 Aurelia Hospital, Rome, Italy

**Keywords:** Knee replacement, Arthroplasty, Drainage, Knee arthroplasty

## Abstract

**Introduction:**

Total knee arthroplasty is a commonly performed procedure with postoperative complications including blood loss, pain, and hematoma formation. The role of intra-articular drainage in reducing these complications remains controversial. This study aims to evaluate the effects of intra-articular drainage on hemoglobin levels, pain, hematoma formation, and the need for blood transfusion in patients undergoing knee replacement.

**Materials and Methods:**

A prospective study was conducted on 60 patients divided into two groups: Drainage (D) and No Drainage (ND). Hemoglobin levels, pain scores, ultrasound evaluation of intra-articular hematoma, and transfusion rates were recorded on the first, third, and fifth postoperative days. The estimated blood loss was calculated using the Meunier formula. Pain was evaluated using the Visual Analog Scale, while hematoma size was measured using ultrasound.

**Results:**

The postoperative hemoglobin drop was significantly higher in the D group (*p* = 0.0027), with a mean estimated blood loss of 1366 mL in the D group compared to 957 mL in the ND group. Four patients in the D group required blood transfusions, while none in the ND group did (*p* = 0.0003). Pain scores were lower in the D group on the first postoperative day (*p* = 0.0001), but no significant differences were observed on the third and fifth days. Ultrasound showed a larger hematoma in the ND group on the first postoperative day (*p* = 0.02), but no significant difference was found by the fifth day.

**Conclusion:**

The use of drainage in TKA is associated with increased blood loss and transfusion rates without providing long-term clinical benefits. While it may offer temporary pain relief and reduce early hematoma formation, its routine application should be reconsidered given the potential for increased postoperative anemia and patient discomfort, along with the lack of sustained pain reduction. Surgeons should carefully weigh its short-term benefits against its risks, particularly in patients with pre-existing anemia or those at risk for significant blood loss.

## Introduction

Total knee arthroplasty (TKA) is a highly successful, reproducible, and widely utilized surgical procedure, recognized as one of the most effective orthopedic interventions for treating end-stage osteoarthritis and rheumatic diseases, achieving favorable clinical and functional outcomes [1]. The global demand for TKA is increasing, with projections indicating continued growth beyond 2030 [2]. Consequently, there is an ongoing and intensifying debate regarding the refinement of surgical techniques [3]. Given that TKA involves both soft tissue and bone dissection, several perioperative complications can arise. The orthopedic literature has examined various factors that can influence TKA outcomes, including preoperative planning, implantation techniques, implant materials, and intraoperative and postoperative strategies [4].

This study focuses on the use of postoperative intra-articular suction drainage in total knee replacement. Suction drainage is a commonly employed routine procedure in orthopedic surgery, aimed at preventing hematoma formation, which can lead to increased incision tension, restricted mobilization, impaired wound healing, heightened pain, and a greater risk of infection [5, 6]. Suction drainage may also be associated with the reduction of systemic effects (e.g., fever, blood loss) and local effects (e.g., pain, swelling, wound healing complications, hematoma formation, superficial and deep infections) [7, 8]. However, the actual benefits of drainage in TKA remain controversial [9, 10], with no consensus on its efficacy and poorly established guidelines, despite numerous studies addressing its risks and benefits [11, 12]. Additionally, the presence of drainage introduces a conduit between the articular cavity and the external environment, potentially increasing the risk of retrograde infection [13]. Therefore, this study aims to evaluate the true utility of suction drainage in TKA concerning postoperative bleeding control, pain management, wound complications, and length of hospital stay. This includes an objective assessment of intra-articular hematoma evolution in the early postoperative days through ultrasound measurements. Given the absence of definitive guidelines, the findings of this study could provide valuable insights to refine current surgical protocols and enhance postoperative care.

## Materials and Methods

### Study Design and Setting

This prospective multicenter trial was conducted at the Università Cattolica del Sacro Cuore (UCSC) Policlinico Universitario A. Gemelli in Rome (Italy) and at Mater Olbia Hospital in Olbia (Italy). A total of 60 consecutive patients were enrolled in the study, with 30 patients undergoing surgery at UCSC and 30 at Mater Olbia Hospital between October 1, 2020 and October 1, 2023. Patients meeting the inclusion criteria consented to participate in the study prior to undergoing TKA. Demographic and clinical data were recorded in a computerized database. The sample size was determined using a power analysis based on an expected effect size of 0.5, aiming for a statistical power of 80% and a significance level of 0.05. This calculation indicated that 30 patients per group would be adequate to detect meaningful differences in hemoglobin drop, pain scores, and hematoma formation between the two study arms. The sample size also accounted for potential dropout rates and variability in clinical outcomes.

Inclusion Criteria:

- Patients aged 18 years or older undergoing primary TKA
- Patients who provided written informed consent to participate in the study

Exclusion Criteria:

- Revision surgery
- Rheumatoid arthritis
- History of TKA or deep venous thrombosis
- Knee stiffness
- Coagulation disorders
- Severe comorbidities such as uncontrolled hypertension
- Severe cardiovascular disorders and organ failure
- Patients refusing blood transfusions
- Contraindications to topical tranexamic acid (TXA), including thromboembolic syndrome, coagulopathy, allergy, or major risk of thrombosis
- Infectious diseases
- Neoplastic diseases
- Vasculopathy

The primary indication for TKA was end-stage osteoarthritis with failure of conservative treatments. All patients underwent a preoperative physical examination, and plain radiographs were performed to confirm the diagnosis and assess the severity of osteoarthritis. All surgical procedures were performed by two senior surgeons with extensive experience in joint arthroplasty.

Patients were divided into two groups:

- Drainage (D) Group: Patients who underwent TKA and received postoperative suction drainage
- No Drainage (ND) Group: Patients who underwent TKA without postoperative suction drainage

Patients were randomly assigned to either the Drainage (D) or No Drainage (ND) group using a computer-generated randomization sequence. The allocation was kept secret in sealed, opaque envelopes that were opened in the operating room immediately before surgery. This method ensured an unbiased distribution and reduced selection bias.

Thirty patients were allocated to each group: the Drainage group (D) had a mean age of 72.6 ± 8.2 years (12 males, 18 females) and the No Drainage group (ND) had a mean age of 70.1 ± 7.35 years (13 males, 17 females). At both UCSC and Mater Olbia hospitals, 15 patients were assigned to the ND group and 15 to the D group (Table 1).

**Table 1:** Demographic distribution

Group	Number of patients	Male	Female	Mean age (SD)	UCSC	Mater Olbia
Drainage (D)	30	12	18	72.6 (8.27)	15	15
No Drainage (ND)	30	13	17	70.1 (7.35)	15	15
Total	60	25	35	71.3	30	30

### Surgical Technique

All surgeries were performed under regional anesthesia when feasible by two orthopedic surgeons at Policlinico Universitario A. Gemelli and Mater Olbia Hospital. The primary implant used in the TKA procedures was a cemented fixed-bearing component. Depending on patient-specific indications and surgeon preference, either a posterior-stabilized implant with minimal rotational constraint (Attune, Johnson & Johnson/DePuy, Warsaw, IN, USA) or a cruciate-retaining prosthesis (Optetrak Logic, Exactech, Gainesville, FL, USA) was utilized. Prophylactic antibiotics, specifically 2 g of Cefazolin intravenously, were administered 30 min prior to skin incision.

Patients were positioned supine with the knee flexed at 90°. A midline skin incision of 15 cm and a medial parapatellar approach were employed in all cases. Intra-articular soft tissue balancing, bone measurement, and resection were performed. The prostheses were cemented using the conventional technique with antibiotic-treated cement. A tourniquet was inflated to 100 mmHg above the systolic blood pressure prior to incision and deflated following cementing of the implants. After ensuring hemostasis and closing the wound, a compressive wrap with elastic bandages was applied. Additionally, 1 g of tranexamic acid (Tranex 500 mg/mL; Malesci Spa, Italy) was administered intravenously before deflation of the tourniquet, and 500 mg of tranexamic acid (Tranex 500 mg/mL; Malesci Spa, Italy) was injected locally into the intra-articular space. The wound was irrigated and closed with a compressive dressing. There were no differences in wound closure techniques or dressings between the two groups.

In the Drainage (D) group, a small incision was made along the midline incision to insert an intra-articular suction drain before closing the joint capsule. All patients in the D group received a PVC round drain with negative pressure, which was opened 15 min after tourniquet removal. Drains were removed on the second postoperative day in all 30 cases. All patients received thromboprophylaxis, unless contraindicated, in the form of 4000 IU of enoxaparin calcium administered as subcutaneous injections once daily for 40 days post-surgery or until full weight-bearing was achieved. Both groups were prescribed standard patient-controlled analgesia, including opioids and NSAIDs. All patients received intravenous paracetamol (1 g every 8 h) for 3 days and oral oxycodone (5 mg) if the Visual Analog Scale (VAS) score exceeded 3 (maximum of 3 tablets per day). A physiotherapy protocol, incorporating both active and passive exercises as well as isometric muscle strengthening, was initiated on the first postoperative day for both groups to promote faster recovery and reduce hospital stays.

### Hemoglobin Reduction and Postoperative Blood Loss Evaluation

The primary outcome assessed was the reduction in hemoglobin levels over the first five postoperative days. Complete blood counts were obtained preoperatively and on the first, third, and fifth postoperative days. Blood transfusion was indicated for hemoglobin levels below 8 g/dL or for symptoms, such as tachycardia, tachypnea, and weakness in patients with hemoglobin levels between 8 and 10 g/dL. Estimated blood loss (EBL) was calculated using the Meunier formula, which accounts for changes in hemoglobin levels and patient blood volume [14].

### Clinical Evaluation

Postoperative pain was evaluated using the VAS score, with measurements recorded on the first, third, and fifth postoperative days. Pain levels were compared between the two groups. Additionally, the length of hospital stay was documented and compared between the groups. The incidence of knee swelling, surgical wound bleeding, and wound dehiscence was also assessed and recorded for both groups.

### Ultrasound Evaluation

All patients enrolled in the study underwent follow-up knee ultrasound examinations utilizing a linear multifrequency probe, with multiple scans conducted in both axial and sagittal planes to assess intra-articular hematoma formation following TKA. Specifically, the hematoma in the subquadriceps region was evaluated with the ultrasound probe, measuring both the length and width of the hematoma on the first and fifth postoperative days. Given the operator-dependence of articular ultrasound, all procedures were conducted by a single operator at each hospital. This approach was implemented to ensure consistency and reliability of the examinations and to minimize potential errors.

### Statistical Analysis

The Shapiro–Wilk test was used to assess the normality of the data. For normally distributed data, the Student’s *t*-test was used; for non-normally distributed data, the Mann–Whitney *U* test was applied. ANOVA with Dunnett’s post-hoc test was used for multiple comparisons. Statistical analysis was performed using GraphPad Prism 10 software (San Diego, CA, USA). A *p*-value < 0.05 was considered statistically significant.

### Methodological Limitations

This study presents several methodological limitations that should be acknowledged. Initially, the allocation of patients to the D or ND group was influenced by the routine practices of the operating surgeons at each center, which could have introduced selection bias. However, to mitigate this issue, patients were subsequently randomly assigned to either the D or ND group using a computer-generated randomization sequence. The allocation was kept secret in sealed, opaque envelopes that were opened in the operating room immediately before surgery. This method ensured an unbiased distribution and effectively reduced selection bias. Additionally, while ultrasound provided an objective measure of hematoma size, its operator-dependent nature could introduce variability, despite efforts to standardize assessments. Lastly, the short follow-up period (5 days) limits conclusions on long-term outcomes, such as functional recovery and late complications. Future research with extended follow-up is needed to fully assess the impact of intra-articular drainage in TKA.

## Results

The median age of the patients was 71.3 years (range 48–84), with 72.6 years (SD 8.27) in the Drainage (D) group and 70 years (SD 7.35) in the No Drainage (ND) group (Table 1). In the D group, 17 patients underwent TKA on the right knee and 13 on the left knee. In the ND group, 16 patients had TKA on the right knee and 14 on the left knee.

### Hemoglobin Levels, Estimated Blood Loss and Transfusion Rates

The mean preoperative hemoglobin level was 13.35 g/dL (range 12.88–13.82, SD 1.14) for the D group and 13.12 g/dL (range 12.66–13.58, SD 1.14) for the ND group. In the D group, the mean hemoglobin values recorded on the first, third, and fifth postoperative days were 11.11 g/dL (range 10.64–11.58, SD 1.57), 10.11 g/dL (range 9.68–10.54, SD 1.42), and 9.85 g/dL (range 9.43–10.27, SD 1.37), respectively.

**Fig. 1:**
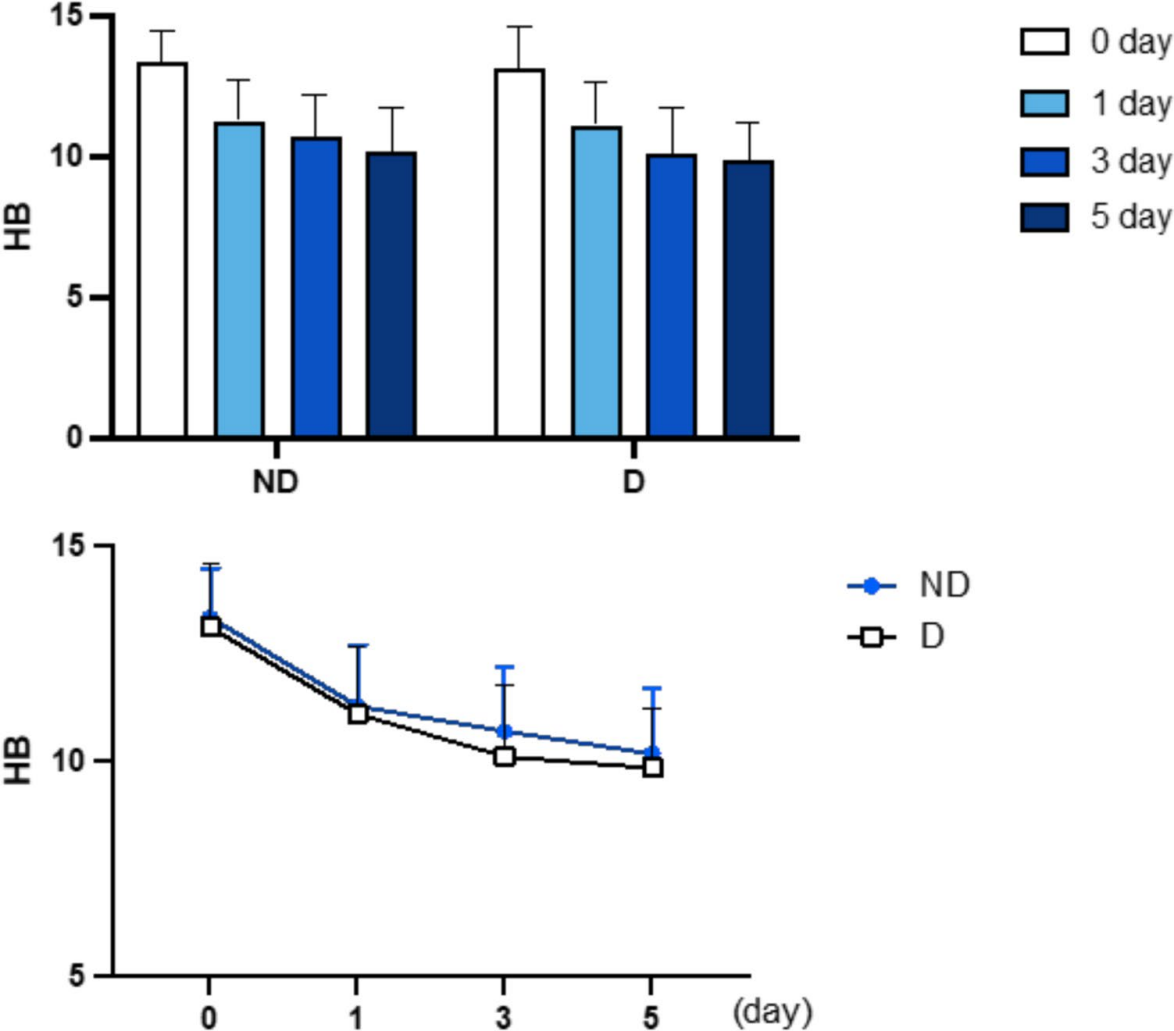
Hemoglobin levels trend

In the ND group, the mean hemoglobin values for the same days were 11.29 g/dL (range 10.84–11.74, SD 1.42), 10.71 g/dL (range 10.32–11.10, SD 1.49), and 10.18 g/dL (range 9.76–10.60, SD 1.52), respectively. Comparisons between the two groups revealed no statistically significant differences in hemoglobin levels on postoperative days 1, 3, and 5, indicating a similar downward trend across measurements (Fig. 1).

For the D group, the mean EBL was 1366 mL (range 1254–1478, SD 351), compared to 957 mL (range 878–1036, SD 278) for the ND group. The greater drop in hemoglobin observed in the D group correlated with a higher EBL. Despite similar preoperative hemoglobin values, the significant reduction in hemoglobin levels in the D group resulted in blood transfusions for four patients (26.6%), whereas none (0%) of the ND group patients required transfusions. The chi-square statistic calculated for transfusion rates between the groups yielded a value of 13.33 (*p* = 0.0003), indicating a statistically significant difference. The Meunier formula further supported the significant difference in hemoglobin reduction between groups (*p* = 0.0027), highlighting the need for careful postoperative monitoring of hemoglobin levels, especially in cases of substantial blood loss.

### Post-operative Pain Evaluation

**Fig. 2:**
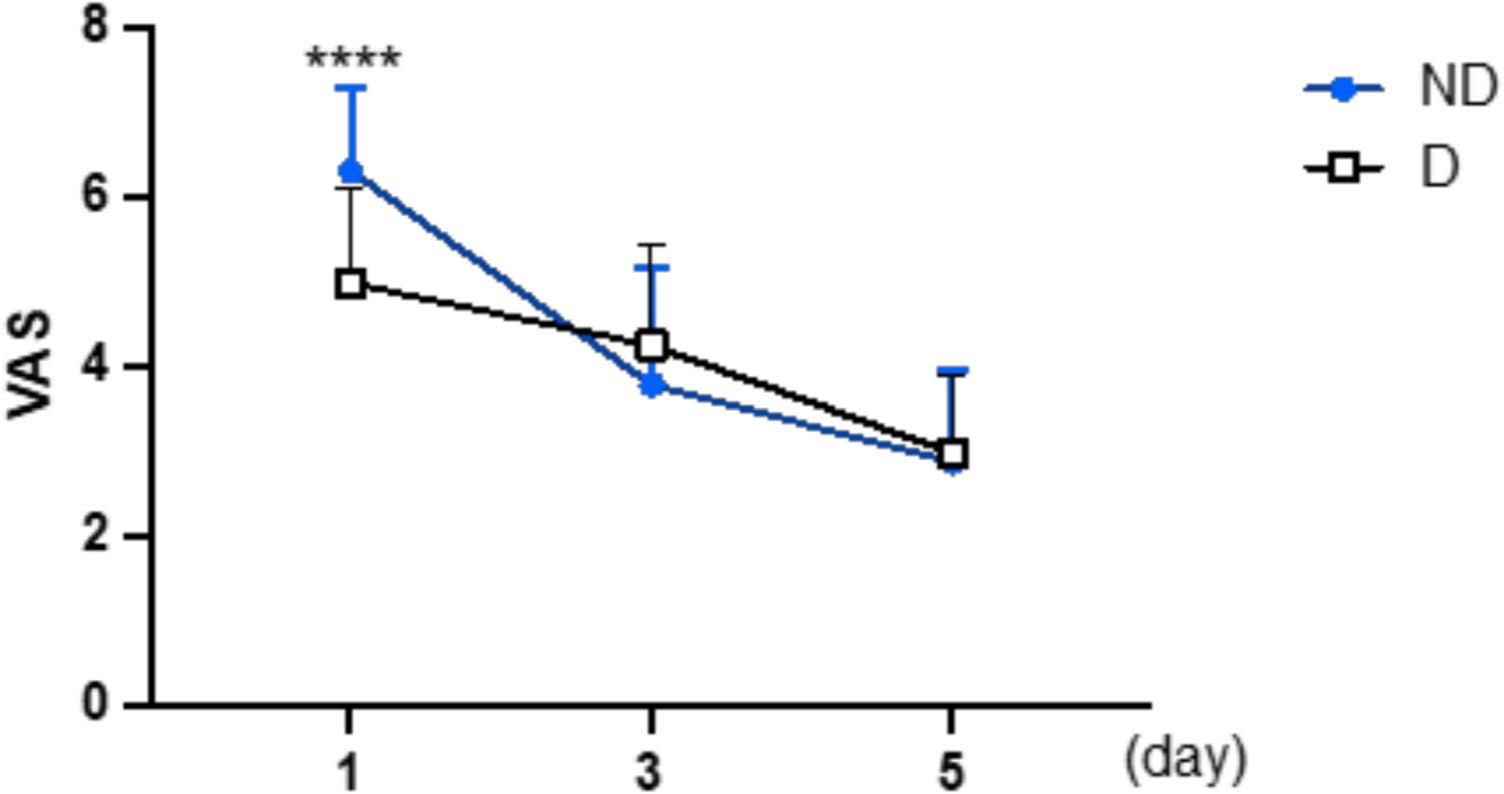
Pain evaluation

Pain levels recorded in the D group showed a mean VAS score of 4.97 (SD 1.13) on the first postoperative day, 4.23 (SD 1.19) on the second day, and 2.97 (SD 0.93) on the fifth day. In the ND group, VAS scores were 6.3 (SD 0.99) on the first postoperative day, 3.77 (SD 1.38) on the second day, and 2.87 (SD 1.07) on the fifth day. On the first postoperative day, pain was statistically significantly lower in the D group (*p* = 0.0001). No statistically significant differences in pain levels were observed on the third (*p* = 0.17) and fifth (*p* = 0.7) postoperative days between the two groups (Fig. 2).

### Hematoma Ultrasound Evaluation

**Fig. 3:**
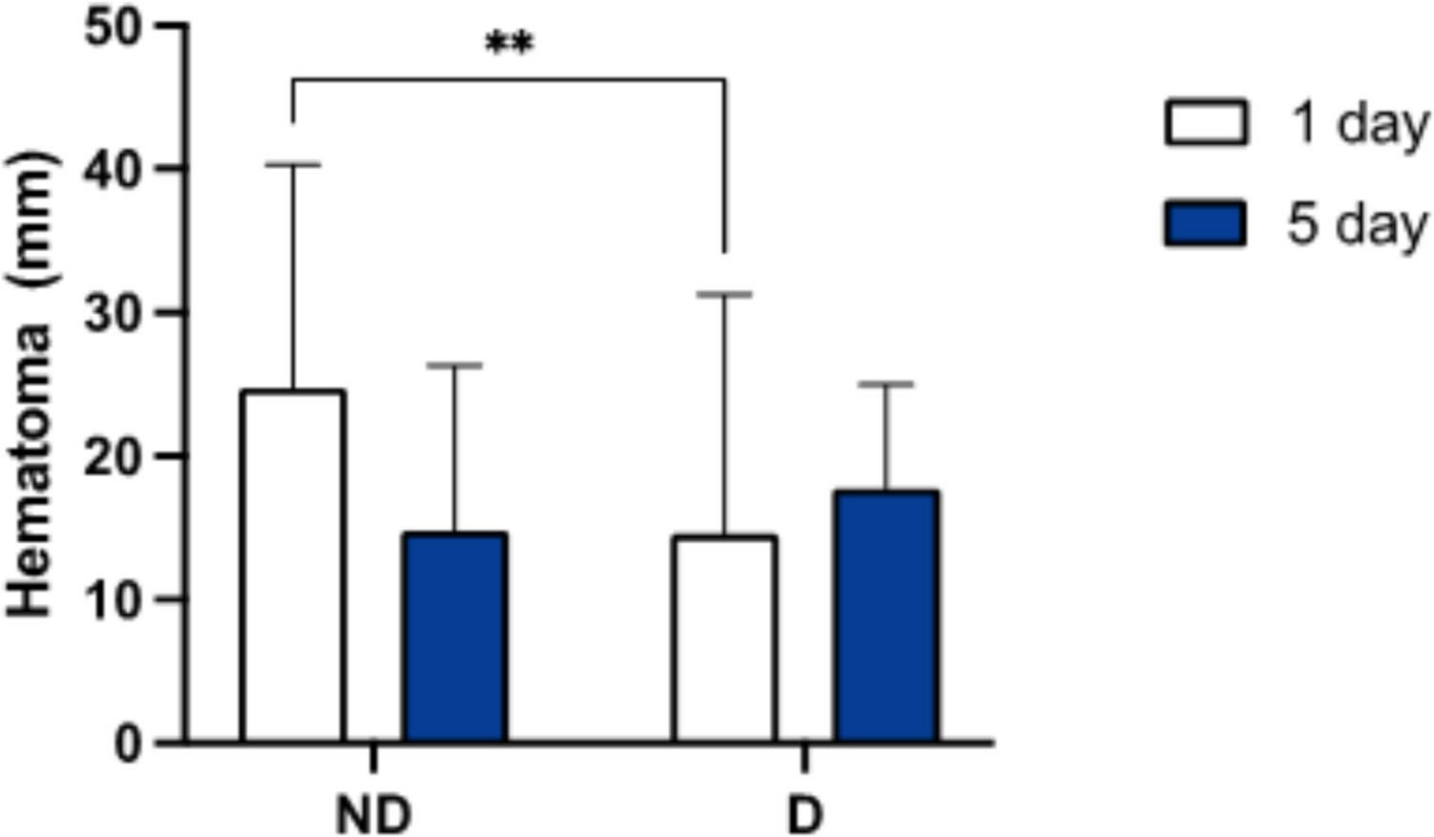
Hematoma evaluation

Ultrasound evaluations of intra-articular hematoma, performed on the first and fifth postoperative days, showed an increasing trend in the D group, from 14.6 mm (range 12.3–16.9, SD 16.6) on the first day to 17.77 mm (range 12.2–17.4, SD 7.02) on the fifth day, after the drain was removed. Conversely, the ND group presented a larger hematoma on the first postoperative day (24.73 mm, range 21.4–28.1, SD 15.58), which was significantly larger compared to the D group (*p* = 0.02). By the fifth postoperative day, the hematoma in the ND group decreased to 14.8 mm (range 12.2–17.4, SD 11.51), with no significant difference from the D group (*p* = 0.46) (Fig. 3).

### Main Hospital Stay Evaluation

**Fig. 4:**
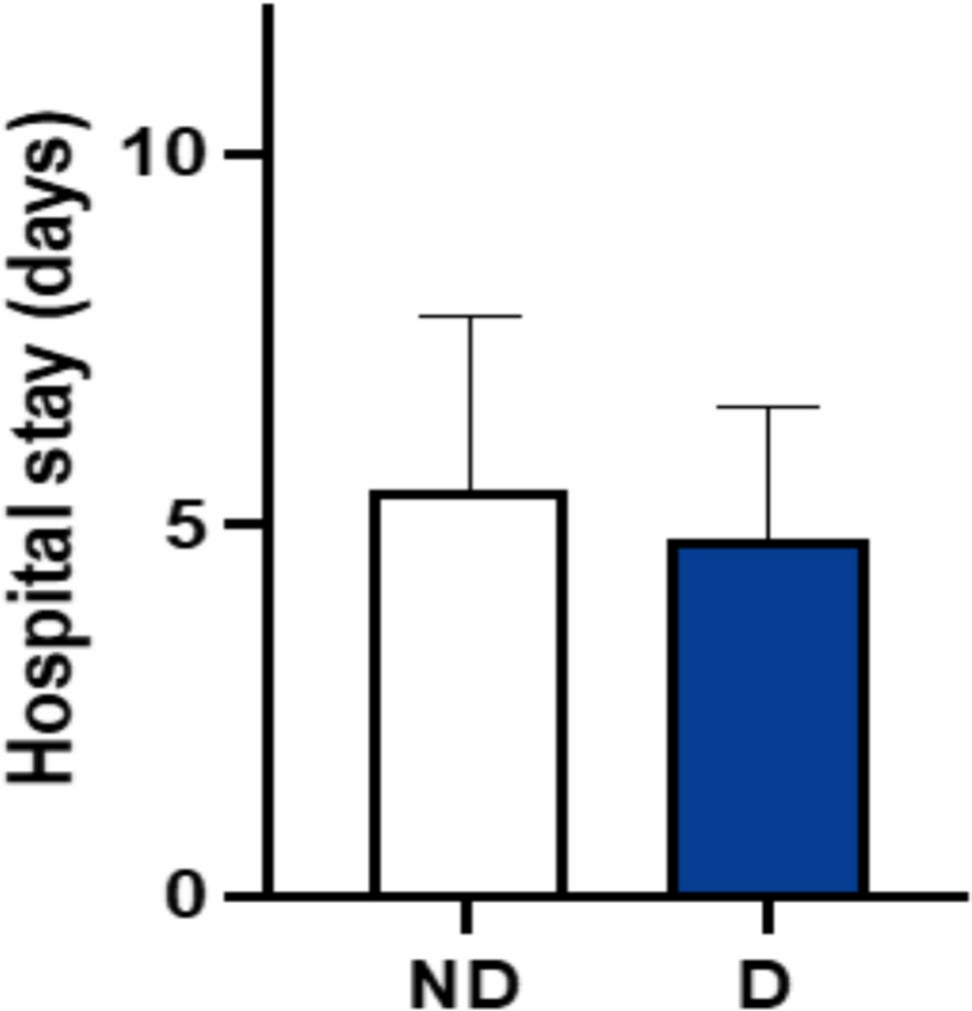
Mean hospital stay evaluation

The mean hospital stay was recorded as 7 days for the D group and 8 days for the ND group. No statistically significant difference was found between the two groups. However, it is important to note that variations in hospital organization may have influenced these data (Fig. 4).

### Qualitative Assessment of Surgical Wounds and Postoperative Complications

A qualitative evaluation of the surgical wounds was performed in both groups throughout the early postoperative period. In the D group, four patients (26.6%) exhibited moderate knee swelling and mild wound bleeding on postoperative days 3 and 4. These patients were closely monitored, and one of them received a 7-day course of oral antibiotics due to mild local erythema. None of the patients in this group required surgical wound revision or demonstrated signs of deep infection. In contrast, no cases of wound bleeding, swelling, or signs of infection were observed in the ND group. Although these findings suggest a trend toward a higher rate of minor wound-related complications in the D group, the differences did not reach statistical significance. No adverse events or major complications, such as deep vein thrombosis, pulmonary embolism, or prosthetic joint infection, were reported in either group during the five-day postoperative follow-up.

## Discussion

### Impact of Intra-articular Drainage on Postoperative Hemoglobin Levels and Blood Management

In this study, we observed that the use of intra-articular drainage in TKA does not lead to significant differences in postoperative bleeding control. The reduction in hemoglobin levels was analyzed to evaluate the extent of anemia associated with TKA surgery. Effective perioperative blood management is essential for minimizing the need for transfusions and includes preoperative diagnosis, correction of anemia, and intraoperative bleeding control [13]. It is commonly believed that intra-articular drainage may exacerbate postoperative bleeding in TKA. However, the relationship between drainage use and increased blood loss remains ambiguous in the literature. Several studies comparing TKA patients with and without drainage have found no significant difference in hemoglobin drop [15, 16], whereas other studies have reported significant postoperative hemoglobin drops linked to drainage use [17, 18].

Our hypothesis posits that the hematoma formed in the early postoperative period serves as a local compressive-mechanical hemostatic agent, potentially mitigating the typical hemoglobin drop observed in the postoperative days. This hypothesis is supported by our data, which indicate that patients without drainage experienced a smaller reduction in hemoglobin and maintained higher levels throughout the postoperative period compared to those with drainage. Additionally, although not statistically significant, the drainage group had a higher incidence of blood transfusions, with four patients requiring transfusions compared to none in the no-drainage group. These findings suggest that the use of drainage may be associated with an increased risk of postoperative anemia and a higher likelihood of requiring blood transfusions.

### Postoperative Pain and Rehabilitation

Postoperative pain is intricately linked to the patient's overall condition and well-being during the hospital stay. Furthermore, the less pain a patient experiences, the more they can engage with their rehabilitation program following knee surgery. As Peters et al. describe in their work, a new analgesic protocol can facilitate pain-free rehabilitation, thereby accelerating the patient’s return to their activities [19].

Consequently, the pain reported by patients in the postoperative period is considered a measurable, reliable, and cost-effective metric, frequently used in studies to assess the advantages of intra-articular suction drainage in TKA. Many studies comparing pain levels between groups with and without drainage have demonstrated no significant difference, suggesting that drainage does not provide an advantage in terms of postoperative pain. For instance, Mortazavi et al. evaluated the VAS in 106 patients who underwent TKA—half with intra-articular suction drainage and half without—and found no significant difference between the two groups [20].

However, some studies have reported reduced use of painkillers and decreased postoperative pain in patients who received suction drainage. Notably, a study by Erne et al. found that patients with TKA who did not have suction drainage experienced higher pain levels up to 6 weeks post-surgery compared to those who had drainage. Despite this, no differences were observed beyond 6 weeks post-surgery [21].

Our study aligns with Erne’s findings, showing a statistically significant difference in VAS scores between the drainage and no-drainage groups on the first postoperative day. However, this significance diminished by the third postoperative day as no difference was observed thereafter. We recorded higher VAS values in the no-drainage group on the first day, but from the third day onwards, when the drainage was removed, the differences between the groups were no longer apparent.

This observation supports the theory that drainage provides a temporary benefit by reducing intra-articular pressure due to hematoma, but its advantages are limited to the period when the drainage is in place. Once removed, the benefits are lost. Additionally, the VAS is a subjective measure although all patients in this study received the same analgesic therapy.

### Ultrasound Evaluation

A key strength of this study is the use of objective parameters to evaluate the evolution of intra-articular hematoma over the first five postoperative days using ultrasound. The primary function of postoperative intra-articular drainage is to evacuate the knee’s intra-articular cavity, remove the hematoma, theoretically reduce swelling and pain, and thereby accelerate rehabilitation.

The primary parameter analyzed in this study was intra-articular hematoma as assessed by ultrasound on the first and fifth postoperative days. This parameter is infrequently addressed in the literature. Omonbude et al. assessed patients who underwent TKA with and without drainage using ultrasound and found no statistically significant difference between the groups [22]. In contrast, our data revealed a significant difference in hematoma diameter on the first postoperative day, with the drainage group exhibiting higher values. However, this significant difference was not observed on the fifth postoperative day, when the drain had been removed in the drainage group. This suggests that the benefit of drainage in reducing hematoma is limited to the period while the drain is in place. Our findings are consistent with those of Varley et al., who studied the effects of drainage on postoperative hematoma formation in patients undergoing hip surgery after proximal femur fracture and found that drainage was effective in reducing hematoma only while it was positioned within the joint [23].

### Limitations

While the study provides valuable insights, some methodological limitations must be considered. First, selection bias may have influenced the results, as patient allocation was based partly on surgeon preference at each center. Although standard surgical techniques were applied, this variability could have introduced minor differences in perioperative management. Second, ultrasound evaluation of hematoma—while an objective measure—remains operator-dependent, with potential interobserver variability. To mitigate this, assessments were performed by a single trained operator at each hospital, ensuring consistency.

Finally, the follow-up period was limited to the first five postoperative days, meaning that longer-term effects of drainage on functional recovery, late complications, or persistent joint stiffness were not assessed. Future studies with extended follow-up could provide a more comprehensive evaluation of drainage’s impact on rehabilitation outcomes.

## Conclusion

This study found no significant difference in hospital stay between TKA patients with and without suction drainage. While drainage was associated with lower pain levels and smaller hematoma dimensions on the first postoperative day, these benefits disappeared by the third day. Additionally, drainage led to greater hemoglobin loss and a higher transfusion rate, without long-term advantages in pain relief or hematoma control. Patients with drainage also experienced a delay in knee mobilization due to discomfort, further questioning its routine use. Based on these findings, routine use of suction drainage in TKA is not recommended. Given the increased blood loss, higher transfusion rates, and delayed rehabilitation, drainage should be used selectively rather than as a standard practice. Surgeons should consider alternative strategies, such as topical hemostatic agents or closed-suction drainage systems with anti-reflux mechanisms, to minimize blood loss while avoiding unnecessary interventions. Patient-specific factors, such as anticoagulant use or a high risk of hematoma formation, should guide the decision on whether drainage is necessary.

As surgical techniques evolve, rethinking drainage protocols in TKA will be crucial to optimizing recovery and improving patient outcomes. Future research should focus on long-term functional recovery, refining personalized drainage indications, and evaluating alternative blood management approaches to further improve perioperative care.

## Data Availability

The datasets generated during and/or analyzed during the current study are available from the corresponding author on reasonable request for at least 8 years from formal conclusion of this study.
